# Enteric glial cell network function is required for epithelial barrier restitution following intestinal ischemic injury in the early postnatal period

**DOI:** 10.1152/ajpgi.00216.2022

**Published:** 2023-12-26

**Authors:** Amanda L. Ziegler, Madison L. Caldwell, Sara E. Craig, Emily A. Hellstrom, Anastasia E. Sheridan, Melissa S. Touvron, Tiffany A. Pridgen, Scott T. Magness, Jack Odle, Laurianne Van Landeghem, Anthony T. Blikslager

**Affiliations:** ^1^Department of Clinical Sciences, College of Veterinary Medicine, North Carolina State University, Raleigh, North Carolina, United States; ^2^Joint Department of Biomedical Engineering, School of Medicine, University of North Carolina, Chapel Hill, North Carolina, United States; ^3^Department of Animal Science, College of Agriculture and Life Sciences, North Carolina State University, Raleigh, North Carolina, United States; ^4^Department of Molecular Biomedical Sciences, College of Veterinary Medicine, North Carolina State University, Raleigh, North Carolina, United States

**Keywords:** enteric glia, epithelial restitution, intestinal barrier, intestinal ischemia, restitution

## Abstract

Ischemic damage to the intestinal epithelial barrier, such as in necrotizing enterocolitis or small intestinal volvulus, is associated with higher mortality rates in younger patients. We have recently reported a powerful pig model to investigate these age-dependent outcomes in which mucosal barrier restitution is strikingly absent in neonates but can be rescued by direct application of homogenized mucosa from older, juvenile pigs by a yet-undefined mechanism. Within the mucosa, a postnatally developing network of enteric glial cells (EGCs) is gaining recognition as a key regulator of the mucosal barrier. Therefore, we hypothesized that the developing EGC network may play an important role in coordinating intestinal barrier repair in neonates. Neonatal and juvenile jejunal mucosa recovering from surgically induced intestinal ischemia was visualized by scanning electron microscopy and the transcriptomic phenotypes were assessed by bulk RNA sequencing. EGC network density and glial activity were examined by Gene Set Enrichment Analysis, three-dimensional (3-D) volume imaging, and Western blot and its function in regulating epithelial restitution was assessed ex vivo in Ussing chamber using the glia-specific inhibitor fluoroacetate (FA), and in vitro by coculture assay. Here we refine and elaborate our translational model, confirming a neonatal phenotype characterized by a complete lack of coordinated reparative signaling in the mucosal microenvironment. Furthermore, we report important evidence that the subepithelial EGC network changes significantly over the early postnatal period and demonstrate that the proximity of a specific functional population of EGC to wounded intestinal epithelium contributes to intestinal barrier restitution following ischemic injury.

**NEW & NOTEWORTHY** This study refines a powerful translational pig model, defining an age-dependent relationship between enteric glia and the intestinal epithelium during intestinal ischemic injury and confirming an important role for enteric glial cell (EGC) activity in driving mucosal barrier restitution. This study suggests that targeting the enteric glial network could lead to novel interventions to improve recovery from intestinal injury in neonatal patients.

## Graphical abstract

**Figure:**
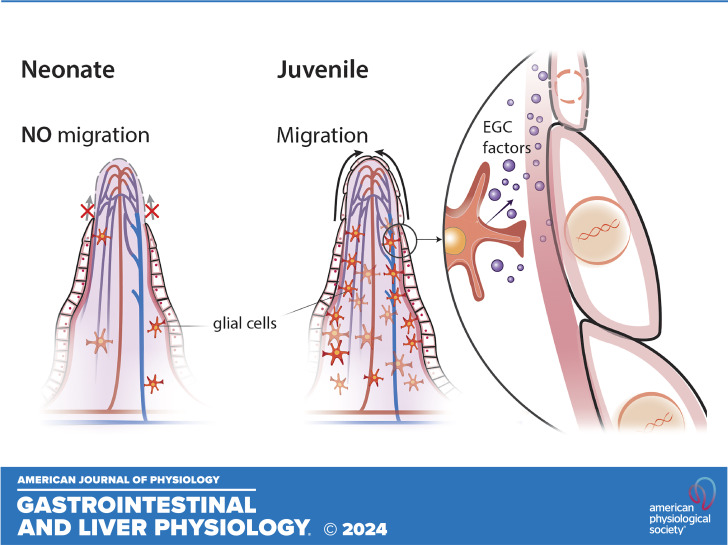

## INTRODUCTION

The intestinal epithelium is sensitive to a range of digestive disease insults, particularly those characterized by ischemia, because of the villus counter-current blood flow that exacerbates epithelial hypoxia at the tips of villi (1, 2). Ischemia-induced breakdown of the intestinal epithelial barrier leads to sepsis, intestinal necrosis, and host death unless the barrier is rapidly and completely repaired following an ischemic event (1–4). Intestinal ischemia is a component of devastating diseases such as strangulating obstruction and neonatal necrotizing enterocolitis (5). For example, a cause of strangulating obstruction in infants, volvulus, most commonly occurs because of anatomical mesenteric abnormalities and, in terms of age, has the highest reported mortality in the immediate newborn period (6). Despite many advances in the management of critical patients with gastrointestinal failure, severe intestinal injuries that impact the intestinal barrier persistently have much worse reported clinical outcomes in younger patient populations. A disease associated with particularly severe barrier dysfunction, necrotizing enterocolitis, is associated with unremittingly high mortality rates between 20% and 30%, with the youngest patients, preterm neonates, suffering the worst outcomes (7, 8). These poorer clinical outcomes in neonates as compared with older patients have not been fully explained, and this represents a critical limitation to our ability to improve survival in these patients.

Clinical interventions for neonatal intensive care unit patients with ischemia-injured intestines currently include surgical correction of the obstruction, supportive care, attention to sepsis that results from disruption of the intestinal barrier, and ultimately intestinal resection when necessary (9, 10). Novel treatments have focused on stimulating de novo formation of new epithelial cells from the stem cell niche, but this requires the support of the patient for days following the initial injury until newly produced epithelial cells can restore the mucosal barrier (9, 11). In the early reparative phase (within hours), the remaining epithelium can immediately restitute the damaged barrier to curtail sepsis early on in the clinical onset of the disease and prevent patient mortality until the regenerative phase can fully restore intestinal architecture (1). For this reason, our laboratory has focused on understanding the mechanisms of the early phase of repair, as clinical interventions enhancing this acute reparative phase are particularly promising for improving patient survival.

We study mechanisms of acute mucosal repair following ischemia using a porcine model because pigs are widely appreciated as one of the most powerful comparative models for human biology and disease, and for gastrointestinal disease in particular, due to many important similarities between porcine and human diet, size, and gastrointestinal physiology (12–17). The vascular anatomy of humans and pigs has been shown to be remarkably similar, with comparable levels of epithelial injury induced by oxidative enzymes and neutrophil infiltration during ischemic events (2, 18). In juvenile (6–8 wk old) pigs, we have shown rapid restitution and recovery of intestinal barrier function, effectively minimizing sepsis and complications in the host after injury. We recently found that in neonatal (2-wk old) pigs, the epithelial barrier failed to restitute under the same conditions (19). An age-dependent defect in epithelial restitution in neonates is likely to be a critical contributor to the high morbidity and mortality seen in infant patients clinically affected by intestinal ischemia and barrier injury. Therefore, this newly identified age-dependent intestinal repair defect in a highly comparative porcine model can provide critical insight for addressing the unacceptably high morbidity and mortality rates suffered by the youngest of critically ill patients. Importantly, we have found that neonatal restitution can be rescued by direct application of ischemic-injured homogenized mucosa from juvenile pigs, suggesting that neonatal epithelial cells are intrinsically capable of efficient restitution in response to yet uncharacterized cues present in juvenile mucosa (19). Therefore the identification of the components of the juvenile mucosal tissues that rescue neonatal restitution will inform future development of novel interventions for devastating intestinal diseases associated with age-dependent defects in barrier repair mechanisms.

A complex and dense population of mesenchymal, circulatory, immune, and neural cells within the lamina propria are known to modulate epithelial cell functions in homeostasis and disease states by secreting paracrine factors to orchestrate complex, coordinated signaling in the mucosal microenvironment. Work in rodents suggests that some of these cell populations are fully developed at birth, whereas others, particularly enteric glia, develop postnatally to form a dense network in close proximity to intestinal epithelial cells. This glial network continues maturation before and after weaning during the postnatal period in rodents (20–22). In addition, a growing body of evidence indicates that enteric glial cells (EGCs) play a pivotal role in promoting intestinal epithelial barrier function and healing through paracrine signaling mechanisms (23–28). This led us to hypothesize that the developing glial network may play an important role in the establishment of efficient mechanisms of intestinal barrier repair during the postnatal period. In this study, we sought to examine how the subepithelial enteric glial network changes over the early postnatal period and how these cells impact intestinal barrier restitution following ischemic injury using our porcine model.

## MATERIALS AND METHODS

### Animals

All procedures were approved by North Caroline State University Institutional Animal Care and Use Committee. Commercial crossbred pigs 2- or 6 wk of age of either sex were euthanized at an academic animal production facility for immediate tissue collection or were transported to a large animal surgical facility for surgical modeling experiments.

### Surgical Ischemic Injury Model

Pigs were sedated using xylazine (1.5 mg/kg) and ketamine (11 mg/kg). Anesthesia was induced with isoflurane vaporized in 100% oxygen via face mask, after which pigs were orotracheally intubated for continued delivery of isoflurane to maintain general anesthesia. Pigs were placed on a water-circulated heating pad, and intravenous fluids were administered at a maintenance rate of 15 mL/kg/h throughout the surgery. The distal jejunum was accessed via midline or paralumbar incision and 8- to 10-cm loops were ligated in segments and subjected to 30 min of ischemia via ligation of local mesenteric blood vessels with 2-0 braided silk suture. Adjacent loops not subjected to ischemia were used as control tissue. For enteric glial cell inhibition studies, Ringer solution containing 0, 5, 500, or 5,000 µM sodium fluoroacetate (MP Biomedicals, Cat. No. ICN201080) was injected into the lumen of the intestinal loops. At the time of tissue collection, pigs were euthanized with an overdose of pentobarbital. Intestinal loops were excised and opened longitudinally along the antimesenteric border and placed in a cold, oxygenated Ringer solution. For enteric glial cell inhibition studies, tissues were transported from the surgical facilities to the laboratory for Ussing chamber experiments in cold, oxygenated Ringer solution containing 0, 5, 500, or 5,000 µM sodium fluoroacetate.

### Scanning Electron Microscopy

Thirty-minute ischemic neonate and juvenile jejunum were fixed at 30- and 120-min during the ex vivo recovery period in independent experiments. Mucosa was rinsed briefly with PBS to remove surface debris followed by immersion fixation in 2% paraformaldehyde-2.5% glutaraldehyde-0.15 M sodium phosphate buffer, pH 7.4. Specimens were stored in the fixative overnight for several days at 4°C before processing for scanning electron microscopy (SEM) (Microscopy Services Laboratory, Department of Pathology and Laboratory Medicine, UNC, Chapel Hill, NC). After three washes with 0.15 M sodium phosphate buffer (PBS), pH 7.4, the samples were postfixed in 1% osmium tetroxide in PBS for 1 h and washed in deionized water. The samples were dehydrated in ethanol (30%, 50%, 75%, 100%, 100%), transferred to a Samdri-795 critical point dryer, and dried using carbon dioxide as the transitional solvent (Tousimis Research Corporation, Rockville, MD). Tissues were mounted on aluminum planchets using silver paste and coated with 15 nm of gold-palladium alloy (60Au:40Pd, Hummer X Sputter Coater, Anatech USA, Union City, CA). Images were taken using a Zeiss Supra 25 FESEM operating at 5 kV, using the SE2 detector, 30-µm aperture, and a working distance of 10–12 mm (Carl Zeiss Microscopy, LLC, Peabody, MA).

### RNA Sequencing Analysis

Jejunal mucosal scrapings were snap-frozen in liquid nitrogen and stored at −80°C until further processing. mRNA was isolated using the RNeasy Mini Kit (Qiagen, Hilden, Germany) and cDNA libraries were generated using SMARTer Stranded Total RNA Sample Prep Kit—HI Mammalian (Takara Bio, Inc., Kusatsu, Japan). Libraries were high-output single-end sequenced on a NextSeq500 instrument (Illumina, Inc., San Diego, CA) for 75 cycles using an 8-base pair index. High-quality Sequences were aligned to an annotated porcine genome (Sscrofa11.1, University of California, Santa Cruz, CA). Before analysis all nontranscribed genes were removed, and all genes that had more than one count per million in two or more samples were analyzed.

### Gene Expression Analysis

With the use of raw counts data from NextSeq500, differential gene expression analyses between neonatal and juvenile injured and uninjured mucosal scrapings were performed using R software (R Foundation for Statistical Computing, Vienna, Austria) with the DESeq2 package (Bioconductor) and RStudio software (RStudio, Boston, MA) to obtain log-fold change (logFC), false discovery rates (FDR), and *P* values as well as generate principal component analysis plots, volcano plots, and lists of top differentially expressed (DE) genes (29–31).

### Pathway and Gene Set Analysis

DE genes identified by DESeq2 in pairwise comparisons were analyzed with Ingenuity Pathways Analysis (Qiagen, Hilden, Germany) to define global regulation of key “Diseases and Functions” pathways related to epithelial cell migration, adhesion, and intestinal injury and barrier function. These functions included “Cell movement,” “Migration of cells,” “Cell survival,” “Activation of cells,” “Organization of cytoskeleton,” “Cellular homeostasis,” “Microtubule dynamics,” “Organization of cytoplasm,” “Inflammation of organ,” “Cell-cell contact,” “Formation of cell protrusions,” “Formation of filaments,” and “Formation of cytoskeleton.”

As a complementary approach, Gene Set Enrichment Analysis (GSEA) was used to predict pathways and functions differentially activated between the experimental groups by determining whether predefined gene sets showed significant enrichment scores when comparing expression profiles of experimental groups. “Hallmark” and “Reactome” gene sets and the following selected Gene Ontology Biological Processes (GO BP, http://geneontology.org/) gene sets were assessed: “Glial Cell Development,” “Glial Cell Proliferation,” “Glial Cell Differentiation,” and “Glial Cell Migration” between age groups and injury groups. Finally, a gene set defining “Gliosis” reported by Schneider et al. (32) in 2021 was interrogated using GSEA.

### Western Blot Analysis

Distal jejunum was rinsed in cold PBS and opened along the antimesenteric border. The tissue was pinned to a transparent silicone-coated glass petri dish mucosal side up under a binocular microscope (National Optical & Scientific Instrument, Inc., Schertz, TX). The mucosa to the level of the muscularis mucosa was microdissected, collected, and snap-frozen in liquid nitrogen until further processing. To isolate proteins, tissues were homogenized using a handheld homogenizer (Omni International, Kennesaw, GA) in T-PER tissue extraction reagent (Thermo Fisher Scientific, Waltham, MA, Cat. No. FNN0071) with Halt Protease Inhibitor Cocktail (Thermo Fisher Scientific, Cat. No. 87785). Samples were centrifuged at 10,000 rpm for 5 min at 4°C to pellet tissue debris. The supernatant was transferred to fresh tubes, and the protein concentrations of the samples were measured with a Pierce BCA protein assay kit (Thermo Fisher Scientific, Cat. No. 23227). Samples were prepared for electrophoresis by denaturing 20 µg of protein per sample in XT Sample Buffer (Bio-Rad, Hercules, CA, Cat. No. 161-0791) and XT Reducing agent (Bio-Rad, Cat. No. 3161-0792) at 95°C for 5 min. Samples were loaded into XT Criterion 4–12% Bis-Tris gels (Bio-Rad, Cat. No. 345-0123) with Precision Plus Protein Dual Color Standards (Bio-Rad, Cat. No. 1610374) and run at 200 V for 35 min in XT MES Running Buffer (Bio-Rad, Cat. No. 161-0789). Gels were transferred to a PVDF membrane (Bio-Rad, Cat. No. 162-0175) at 100 V, 1.5 A for 30 min in 20% methanol Tris/glycine buffer (Bio-Rad, Cat. No. 161-0734). Membranes were blocked in 5% Blotting-Grade Blocker (Bio-Rad, Cat. No. 170-6404) in Tris-buffered saline buffer (Bio-Rad, Cat. No. 170-6435) with 0.01% Tween 20 (TBS/T) for 1 h at room temperature. Membranes were incubated in the following primary antibodies overnight at 4°C: polyclonal rabbit anti-β-actin IgG (Abcam, Cat. No. ab8227, RRID:AB_2305186) at a dilution of 1:1,000, polyclonal rabbit anti-glial fibrillary acidic protein (GFAP) IgG (Agilent, Cat. No. Z0334, RRID:AB_10013382) at a dilution of 1:1,000 in 5% Blotting-Grade Blocker in TBS/T or 5% bovine serum albumin in TBS/T. Membranes were then incubated in horseradish peroxidase (HRP)-conjugated goat anti-rabbit IgG (Thermo Fisher Scientific, Cat. No. 32460) in 5% Blotting-Grade Blocker. Blots were activated with Pierce ECL Substrate (Bio-Rad, Cat. No. 170-5060) and imaged with a Chemidoc imaging system (Bio-Rad, Cat. No. 170-8280). Membranes were stripped between each probe with Restore PLUS Western Blot Stripping Buffer (Thermo Fisher Scientific, Cat. No. 46430). Band densities were measured with Image J software (NIH, Bethesda, MD) and normalized to β-actin density as a sample loading control for quantification.

### Ussing Chamber Studies

The outer seromuscular layer was stripped by blunt dissection in a cool, oxygenated Ringer solution. Stripped jejunal tissue was mounted in 1.12-cm^2^ aperture Ussing chambers. The tissues were bathed in 10 mL warmed, oxygenated (95% O_2_-5% CO_2_) Ringer solution on the basolateral and apical sides. For enteric glial cell inhibition studies, Ringer solution was treated with 0, 5, 500, or 5,000 µM sodium fluoroacetate on both the basolateral and apical sides. Basolateral Ringer solution also contained 10 mM glucose whereas the apical Ringer solution was osmotically balanced with 10 mM mannitol. Bathing solutions were circulated in water-jacketed reservoirs and maintained at 37°C. The spontaneous potential difference (PD) was measured using Ringer-agar bridges connected to calomel electrodes, and the PD was short-circuited through Ag-AgCl electrodes with a voltage clamp that corrected for fluid resistance. Resistance (Ω·cm^2^) was calculated from spontaneous PD and short-circuit current (*I*_sc_). If the spontaneous PD was between −1 and 1 mV, the tissues were current clamped at ±100 µA and the PD rerecorded. *I*_sc_ and PD were recorded every 15 min for 120 min. From these measurements, transepithelial electrical resistance (TEER) was calculated.

### Light Microscopy and Histomorphometry

Tissues were fixed for 18 h in 10% formalin at room temperature immediately following ischemic injury or after 120-min ex vivo recovery period. Formalin-fixed tissues were transferred to 70% ethanol and then paraffin-embedded, sectioned (5 µm), and stained with hematoxylin and eosin for morphological and morphometric analyses. For morphometric analysis of villus injury, the base of the villus was defined as the opening of the neck of the crypts and the height of epithelialization, total height, and width of villus were measured using Image J Software (NIH, Bethesda, MD). The surface area of the villus was calculated as previously described using the formula for the surface area of a cylinder modified by subtracting the base of the cylinder and adding a factor that accounted for the hemispherical shape of the villus tip (33, 34). The percentage of villus epithelialization was used as an index of epithelial injury and restitution.

### Immunofluorescent Histology

Mucosal samples were trimmed and fixed for 18 h in 4% paraformaldehyde (PFA) in PBS at 4°C. Fixed tissues were transferred to 10% sucrose, then 30% sucrose in PBS for 24 h each for cryopreservation. The tissues were then embedded in optimal cutting temperature (OCT) compound and sectioned (7 µm) onto positively charged glass slides for immunostaining. Sections were rehydrated three times in PBS before permeabilization in 0.3% Triton-X in PBS for 20 min. After permeabilization, slides were washed twice in PBS and then blocked (Agilent, Cat. No. S0909) for 1 h at room temperature. Slides were incubated overnight at 4°C in rabbit anti-GFAP (Agilent, Cat. No. Z0334, RRID:AB_10013382) at a dilution of 1:2,000 in antibody diluent (Agilent, Cat. No. S0809). After being washed three times in PBS, slides were incubated for 1 h at room temperature in the dark in goat anti-rabbit conjugated to Alexa Fluor 647 (Molecular Probes, Cat. No. A-21245, RRID:AB_141775) diluted 1:500 in diluent (Agilent, Cat. No. S0809). Tissues were counterstained with the nuclear marker 4′,6-diamidino-2-phenylindole (DAPI, Invitrogen, Cat. No. D1306) at a dilution of 1:1,000 in diluent (Agilent, Cat. No. S0809) at room temperature. Slides were washed three times in PBS and then mounted under a coverslip in aqueous mounting medium (Agilent, Cat. No. S3025). Images were captured using an inverted fluorescence microscope (Olympus IX81, Tokyo, Japan) with a digital camera (ORCA-flash 4.0, Hamamatsu, Japan) using a ×20 objective lens (LUC Plan FLN, Olympus, Tokyo, Japan). One tissue per treatment was incubated with a secondary antibody mixture but no primary antibody mixture serving as negative controls to ensure primary antibody specificity, and specificity was further validated by confirming expected histological distribution patterns and positive staining in purified cultures of pig enteric glia in vitro (see *Immunocytochemistry*). Immunofluorescence intensity was quantified using ImageJ software. Enteric glial cells of the submucosal ganglia were identified in each individual ganglion by expression of glial fibrillary acidic protein (GFAP) costaining with DAPI. GFAP fluorescence signal was quantified for five ganglia per individual (3 individuals per treatment, *n* = 15 ganglia per treatment). Ganglia were outlined using the magic wand tool at a threshold of 18. Three background regions with no visible fluorescence were randomly selected and measured for each ganglion. Corrected total cell fluorescence (CTCF) was calculated for each ganglion as CTCF = Integrated density of glia – (Area of glia × mean gray value of background regions). The above protocol was repeated costaining with rabbit anti-GFAP (Agilent, Cat. No. Z0334, RRID:AB_10013382) at a dilution of 1:1,000 and murine anti-porcine IL6 (R&D systems, No. MAB686) at a reconstitution of 0.5 mg/mL.

### iDISCO Volume Imaging and Quantification

EGC network was labeled and visualized in three dimensions using the immunolabeled three-dimensional imaging of solvent-cleared organs (iDISCO)+ method adapted to intestinal specimens as previously published by our group (35). Antibodies for immunolabeled three-dimensional (3-D) imaging of solvent-cleared organs (iDISCO) analysis were validated for use with methanol by cryosectioning 20-µm sections of PFA fixed and frozen jejunum onto glass slides, incubating the sections for 3 h in 100% methanol, rehydrating the sections in PBS, and proceeding with standard immunostaining protocol described earlier as recommended by the iDISCO method (https://idisco.info/idisco-protocol/). One tissue was incubated with a secondary antibody mixture but no primary antibody mixture serving as negative controls to ensure primary antibody specificity, and specificity was further validated by confirming expected histological distribution patterns (see *Immunofluorescence*). Full-thickness sections of jejunum were rinsed well in cold PBS and fixed in 4% PFA in PBS at 4°C overnight. Once fixed, tissues were trimmed to 3 mm × 5 mm pieces and processed for solvent clearing according to established protocols (36). Samples were dehydrated gradually in methanol, cleared in 66% dichloromethane-33% methanol overnight at room temperature, then bleached with 5% hydrogen peroxide in methanol overnight at 4°C. Samples were then rehydrated gradually and incubated in PBS/Triton-X-glycine/dimethyl sulfoxide (DMSO) solution for 2 days at 37°C and then blocked in PBS/Triton-X-donkey serum/DMSO for 2 days at 37°C. Samples were incubated in methanol-validated polyclonal rabbit anti-GFAP IgG (Agilent, Cat. No. Z0334, RRID:AB_10013382) at a dilution of 1:1,000 in PBS/Triton-X/donkey serum-DMSO solution for 4 days at 37°C. After being washed, samples were incubated in methanol-validated anti-rabbit AlexaFluor 647-conjugated secondary antibody (Thermo Fisher Scientific, Cat. No. A-21245, RRID:AB_2535813) for 4 days at 37°C. Samples were dehydrated as earlier with additional 100% dichloromethane washes and cleared in dibenzyl ether. Samples were stored in dibenzyl ether until imaging with a Lavision Ultramicroscope II light-sheet system (Lavision BioTech, Bielefeld, Germany).

Data were visualized and analyzed using Imaris Image Analysis Software (Bitplane AG, Zurich, Switzerland). To quantify subepithelial EGC density within the mucosa, five villi were randomly selected for each sample after viewing in slice mode in Imaris to ensure that the villi could be clearly visualized to the depth of the muscularis mucosa using autofluorescence of tissue landmarks. The entire villus and the area just beneath its footprint were masked manually using the click to draw surface features from villus tip to the level of the muscularis mucosa. Using the surfaces feature, a surface was interpolated from these hand-drawn bounds and the total villus volume was recorded from the statistics tab for each villus. After manual villus structure isolation, the surfaces wizard was used to manually optimize an algorithm that best identified and marked the true GFAP signal in each villus. The surfaces wizard was then applied to each masked villus structure without additional adjustment to obtain signal volume measurements from the statistics tab. These values were then used to calculate the percent GFAP signal by volume within the lamina propria of each villus, corresponding to the density of EGC within the lamina propria of each villus.

### EGC Isolation and Culture

Twenty-four-well tissue culture plates were coated overnight at room temperature with 0.5 mg/mL poly-l-lysine (Sigma, Cat. No. P2636) in 0.5 M borate buffer and then washed twice with sterile PBS. Jejunal layers containing either submucosal or longitudinal muscle myenteric plexus were microdissected from noninjured pigs. Tissues were next enzymatically dissociated in 1 mg/mL protease (Sigma, Cat. No. P4630) and 0.25 mg/mL collagenase (Sigma, Cat. No. 9891). After enzyme inactivation and additional mechanical dissociation, cell preparations were strained sequentially through 100-µm, 70-µm, and 40-µm cell strainers to enrich for single cells. Cells were next seeded onto fresh poly-l-lysine-coated tissue culture plates and maintained in DMEM/F12 medium supplemented with 0.5% heat-inactivated FBS, 1× GlutaMAX (Gibco, Cat. No. 35050061), 1× B27(Gibco, Cat. No. 17504044), 1× N2 (Gibco, Cat. No. 17502048), and 1× G5 (Gibco, Cat. No. 17503012) supplements to enrich cultures for glial cells. Cells were passaged with 0.01% trypsin in PBS at 80% confluence. EGC was used for all coculture experiments in *passage 1*.

### Immunocytochemistry

First passage EGC cultures were fixed at ∼80% confluency in 4% paraformaldehyde in PBS (Santa Cruz, Cat. No. sc-281692) overnight at 4°C. Fixed cultures were rinsed three times in 1× PBS and maintained in 0.1% sodium azide (Sigma-Aldrich, Cat. No. S2002) in 1× PBS. Cells were permeabilized and saturated for 30 min at room temperature in PBS with 0.01% Triton-X PBS and 4% donkey serum and incubated overnight at 4°C with chicken anti-GFAP (Abcam, ab4674), mouse anti-Sox10 (Santa Cruz, sc-365692), and rabbit anti-S100β (Dako, IR504) antibodies with anti-GFAP and anti-Sox10 diluted 1:1,000 and 1:100 in the Ready-To-Use anti-S100β, respectively. For the contaminants staining, cells were permeabilized and saturated for 30 min at room temperature in PBS with 0.01% Triton-X PBS and 4% donkey serum and incubated overnight at 4°C with chicken anti-Pgp9.5 (Abcam, ab72910), mouse anti-α smooth muscle actin (αSMA) (Abcam, ab7817), and rabbit anti-S100β (Dako, IR504) antibodies with anti-Pgp9.5 and anti-αSMA diluted 1:300 and 1:500 in the Ready-To-Use anti-S100β, respectively. Primary antibodies were removed by rinsing three times with 1× PBS. Cells were then incubated in Alexa Fluor 488, Alexa Fluor 594, and Alexa Fluor 647-conjugated secondary antibodies against chicken (Thermo Fisher Scientific, Cat. No. A-11041, RRID:AB_ 2534098), mouse (Thermo Fisher Scientific, Cat. No. A-21202, RRID:AB_ 141607), and rabbit (Thermo Fisher Scientific, Cat. No. A-21245, RRID:AB_ 2535813) at a 1:500 dilution in 0.01% Triton-X PBS and 4% donkey serum for 2 h at room temperature. Cell nuclei were stained with DAPI (Invitrogen, Cat. No. D1306) at a dilution of 1:1,000 in PBS for 5 min at room temperature. DAPI staining was rinsed three times with 1× PBS, and stained cells were maintained in 0.1% sodium azide in 1× PBS. Images were captured using an inverted fluorescence microscope (Olympus IX81, Tokyo, Japan) with a digital camera (ORCA-flash 4.0, Hamamatsu, Japan) using a ×20 objective lens (LUC Plan FLN, Olympus, Tokyo, Japan). Color channels were merged and recolorized in ImageJ software (NIH, Bethesda, MD) to visualize overlay images.

### IPEC-J2 Cell Scratch Wound Assay

IPEC-J2 cells (an intestinal epithelial cell line originally isolated from the jejunum of a neonatal pig) were cultured, seeded onto Transwell inserts (Corning, Cat. No. 3470), and maintained in DMEM/F12 supplemented with 10% heat-inactivated FBS, 5 ng/mL insulin-transferrin/selenium supplement (Gibco, Cat. No. 41400045), and 5 ng/mL recombinant human EGF (Gibco, Cat. No. PHG0311). Once IPEC-J2 reached confluence, transwell inserts were transferred to wells containing EGC primary cultures (*passage 1*) or blank wells containing only EGC culture medium. After 24 h, scratch wounds were inflicted manually with a disposable 200-µL micropipette tip. Wounds were imaged at 0-, 2-, 4-, and 6-h after scratch wounding using an inverted microscope (Olympus IX81, Tokyo, Japan) with a digital camera (ORCA-flash 4.0, Hamamatsu, Japan) using a ×20 objective lens (LUC Plan FLN, Olympus, Tokyo, Japan) and plate mapping software (CellSens, Olympus, Tokyo, Japan). Percent wound healing was calculated after measuring the total wound area using ImageJ software (NIH, Bethesda, MD).

To test for off-target effects of fluoroacetate (FA) on epithelial wound healing in vitro, IPEC-J2 monocultures were maintained on transwells as described earlier and the media in the basolateral chamber was removed and replaced with 500 µM sodium fluoroacetate diluted in 0.5% FBS EGC media + growth factors (MP Biomedicals LLC, Cat. No. 201080) or fresh 0.5% FBS EGC media + growth factors for untreated controls. The 500 µM sodium fluoroacetate diluted in media was filtered through a 0.2-µm PES filter (Thermo Fisher Scientific, Cat. No. 725-2520) before dosing. Immediately after dosing was completed, IPEC-J2 monolayers were imaged before and after infliction of mechanical scratch wounds with disposable 200-µL micropipette tips. Wounds were imaged at 0, 2, 4, and 6h after scratch wounding using an inverted microscope (Olympus IX81, Tokyo, Japan) with a digital camera (ORCA-flash 4.0, Hamamatsu, Japan) using a ×10 objective lens (LUC Plan FLN, Olympus, Tokyo, Japan) and plate mapping software (CellSens, Olympus, Tokyo, Japan). Percent wound healing was calculated after measuring the total wound area using ImageJ software.

### Statistical Analysis

Data were analyzed using Prism statistical software (GraphPad, La Jolla, CA). TEER and histomorphometry data were reported as means ± SE for a given number (*n*) of animals for each experiment. Results were analyzed by unpaired Mann–Whitney test for nonparametric data, two-way ANOVA (or mixed-model on datasets with missing data points), or two-way ANOVA on repeated measures. For analyses where significance was detected by ANOVA, Sidak’s test was utilized for post hoc pairwise multiple comparisons. The α-level for statistical significance was set at *P* < 0.05. CTCF data are presented as boxplots indicating the minimum, lower quartile, median, upper quartile, and maximum corrected total cell fluorescence values for each treatment group. A Wilcoxon test for nonparametric data was conducted to compare the distribution of CTCF values between the four conditions. *P* < 0.05 was considered significant. The Grubb’s test was used to detect and remove statistical outliers (greater than two times standard deviations from the mean) from datasets.

### Access to Data

All authors had access to all data and have reviewed and approved the final manuscript. Complete raw Illumina FASTQ files are used and pairwise differential expression data for RNA sequencing analyses are deposited into Gene Expression Omnibus (Series Entry GSE212533; https://0-www-ncbi-nlm-nih-gov.brum.beds.ac.uk/geo/query/acc.cgi?acc=GSE212533) for public access.

## RESULTS

### A Restitution Phenotype in Epithelial Cells Fails to Initiate to Repair Ischemia-Induced Mucosal Wounds in Neonatal Intestinal Mucosa

**Figure 1.**
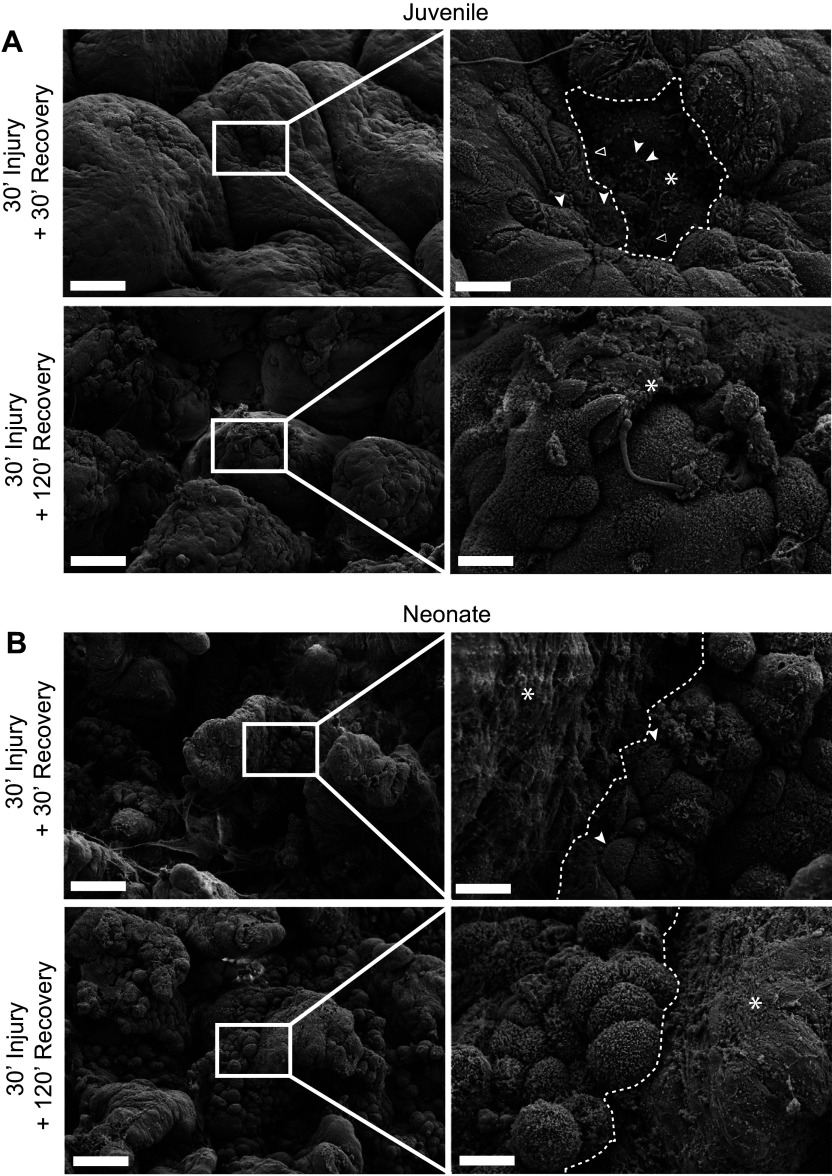
Scanning electron microscopy reveals a persistent epithelial defect in neonatal pigs characterized by a lack of migratory phenotype in the wound adjacent epithelial cells observed in juveniles. *A*: at 30-min ex vivo recovery (*top*), juvenile pigs demonstrate migratory phenotype in wound-adjacent epithelial cells at the wound (asterisk) margins (dashed line). Note the flattening cellular morphology and recruitment of redundant cell membranes from microvilli into the extending lamellipodia (solid arrowhead) and filopodia (open arrowhead). After 120 min of ex vivo recovery (*bottom*), the wound is completely closed, and newly restituted epithelium is visible at the villus tip (asterisk). *B*: at 30 min ex vivo recovery (*top*), neonatal pigs demonstrate a large wound bed (asterisk, border marked with dashed line) surrounded by inactive wound-adjacent epithelial cells (solid arrowhead) that fail to form flattened morphology or migratory projections. After 120 min of ex vivo recovery (*bottom*), the wound bed persists (asterisk, border marked with dashed line) and has enlarged, indicative of further lost cell adhesion and sloughing. Low magnification (*left*) ×1,000, scale bar 40 µm. High magnification (*right*) ×5,000, scale bar 8 µm.

To evaluate and define the age-dependent repair phenotype of small intestinal epithelium, we examined the surface ultrastructure of epithelial wound margins in recovering neonatal and juvenile jejunum by scanning electron microscopy (Fig. 1). At 30-min ex vivo recovery (Fig. 1*A*, *top*), juvenile pigs demonstrate a distinct migratory phenotype in wound-adjacent epithelial cells at the wound margins (dashed line, wound bed marked with an asterisk). The epithelial cells at the wound margins exhibit flattened morphology and recruit redundant cell membranes from microvilli for incorporation into the extending lamellipodia (solids arrowheads) and filopodia (open arrowheads) as the cells migrate across the exposed basement membrane. Later in recovery (at 120 min, *bottom*), the juvenile wounds are completely closed, and newly restituted epithelium, with its characteristic flattened and overlapping morphology, is visible at the villus tip (asterisk). In neonatal pigs after 30 min ex vivo recovery, a large wound bed (Fig. 1*B*, dashed line) is surrounded by inactive wound-adjacent epithelial cells (closed arrowhead), which fail to form flattened morphology or projections characteristic of the migratory phenotype. After 120 min of ex vivo recovery (*bottom*), the neonatal wound bed persists (dashed line, *right*). These images reveal that morphological changes associated with restitution are absent in neonatal mucosa beyond the period of time required for juvenile mucosa to restitute effectively.

**Figure 2.**
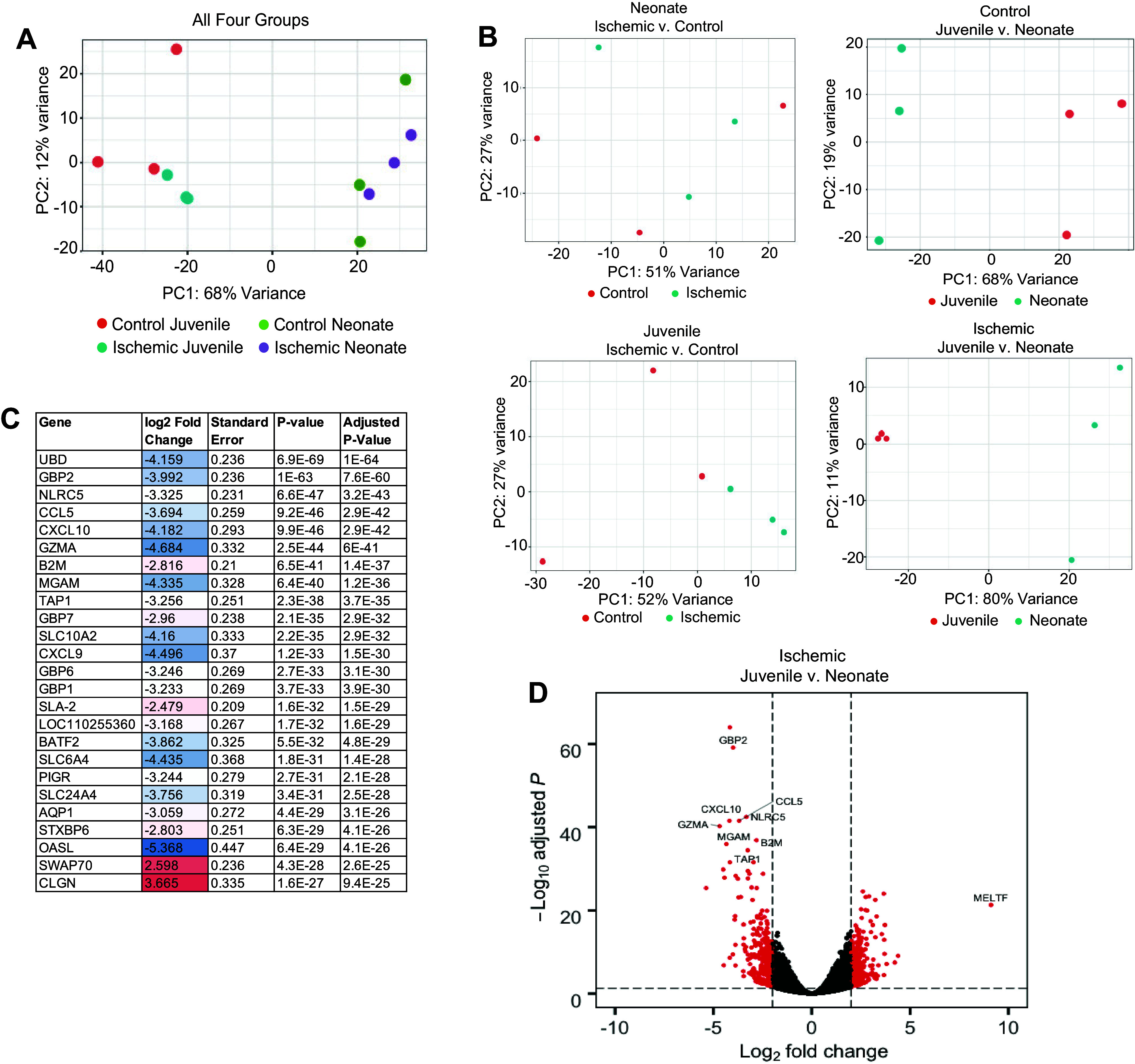
Differential mRNA expression data identifies distinct clustering in response to ischemic injury in juveniles but not neonates. *A*: PCA plot of differentially expressed (DE) genes compared across ischemia-injured or control jejunal mucosa in neonatal and juvenile pigs. Neonates (green and purple) and juveniles (red and blue) cluster distinctly across principal component 1 (PC1) with 68% of variance represented along that axis. Ischemic juveniles (blue) cluster more distinctly from control juveniles (red) than do ischemic neonates (purple) from control neonates (green). *B*: when comparing ischemia effects within age groups, in neonates, there was no distinct clustering between control (red) and ischemic (blue) mucosa (*top left*). Within juveniles, there was clustering of ischemic mucosa (blue) from control mucosa (red) across PC1, which contained 52% of the variance (*bottom left*). When age effects were compared within either control (*top right*) or ischemic (*bottom right*) groups, both control and ischemic juvenile mucosa (red) clustered separately from neonatal mucosa (blue) with 61% and 80% of the variance across PC1, respectively. *C*: top 25 DE genes between ischemic juvenile and ischemic neonatal mucosa are presented. *D*: a volcano plot of DE genes between ischemic juvenile and ischemic neonatal mucosa are presented. (*n* = 3 pigs per age × condition).

To define this phenotype further, we assessed mucosal transcriptional profiles of the neonatal and juvenile jejunum under both ischemic and nonischemic conditions by bulk RNA sequencing. Plotting all four groups revealed distinct age-dependent clustering and a more apparent effect of ischemia in separating the control from ischemic juveniles than control from ischemic neonates (Fig. 2*A*). Plotting only the juvenile groups (548 DE genes) shows clustering of ischemic mucosal samples from control, mostly along the *x*-axis, which accounted for 52% of the total sample variance, indicative of a coordinated transcriptional response to ischemia in juveniles (Fig. 2*B*, *bottom left*). Within neonatal mucosa (no DE genes detected), no appreciable clustering was observed when plotting comparisons of control to ischemic conditions, indicative of a lack of consistent transcriptional response to ischemic injury across the three biological replicates sequenced in this age group (Fig. 2*B*, *top left*). Plotting control tissue (2,313 DE genes) showed distinct clustering of neonatal mucosal samples from juveniles only along the principal component 1 (PC1), with 68% variance along that axis (Fig. 2*B*, *top right*). When age is compared across the ischemic groups (4,862 DE genes), PCA plots demonstrate very tight clustering of ischemic juvenile mucosa distinct from ischemic neonatal mucosa on principal component analysis plots with most of the separation occurring in the *x*-axis accounting for 80% of the total sample variance, indicative of a differing transcriptional response in neonates as compared with juveniles under ischemic conditions (Fig. 2*B*, *bottom right*). A list of the top 25 DE genes between ischemic juvenile and ischemic neonatal tissues (Fig. 2*C*), as well as a volcano plot depicting the genes that drive the distinct clustering of these samples, are presented (Fig. 2*D*). A complete listing of all DE genes for all pair-wise comparisons is publicly available for review in GEO (GSE212533).

The direct comparison of the impact of postnatal age on DE genes under ischemic conditions in a pairwise fashion allowed the use of Ingenuity Pathway Analysis to predict the downregulation of 11 “Diseases and Functions” signaling pathways instrumental in cell migration under ischemic conditions in neonatal mucosa as compared with juveniles (Table 1).

**Table 1.** “Diseases and Functions” Ingenuity Pathways Analysis of differentially expressed gene dataset in ischemic neonatal mucosa relative to ischemic juvenile mucosa

Function	*P* Value	*z* Score	Predicted State
Cell movement	2.26 × 10^−45^	−2.619	↓
Migration of cells	5.10 × 10^−44^	−2.558	↓
Activation of cells	1.38 × 10^−27^	−2.915	↓
Organization of cytoskeleton	3.72 × 10^−26^	−3.067	↓
Cellular homeostasis	2.44 × 10^−24^	−3.014	↓
Microtubule dynamics	1.84 × 10^−22^	−2.543	↓
Organization of cytoplasm	8.67 × 10^−22^	−3.067	↓
Cell-to-cell contact	6.26 × 10^−14^	−3.287	↓
Formation of cell protrusions	8.23 × 10^−14^	−2.825	↓
Formation of filaments	2.27 × 10^−13^	−2.498	↓
Formation of cytoskeleton	3.89 × 10^−13^	−3.013	↓

↓Predicted downregulation.

**Figure 3.**
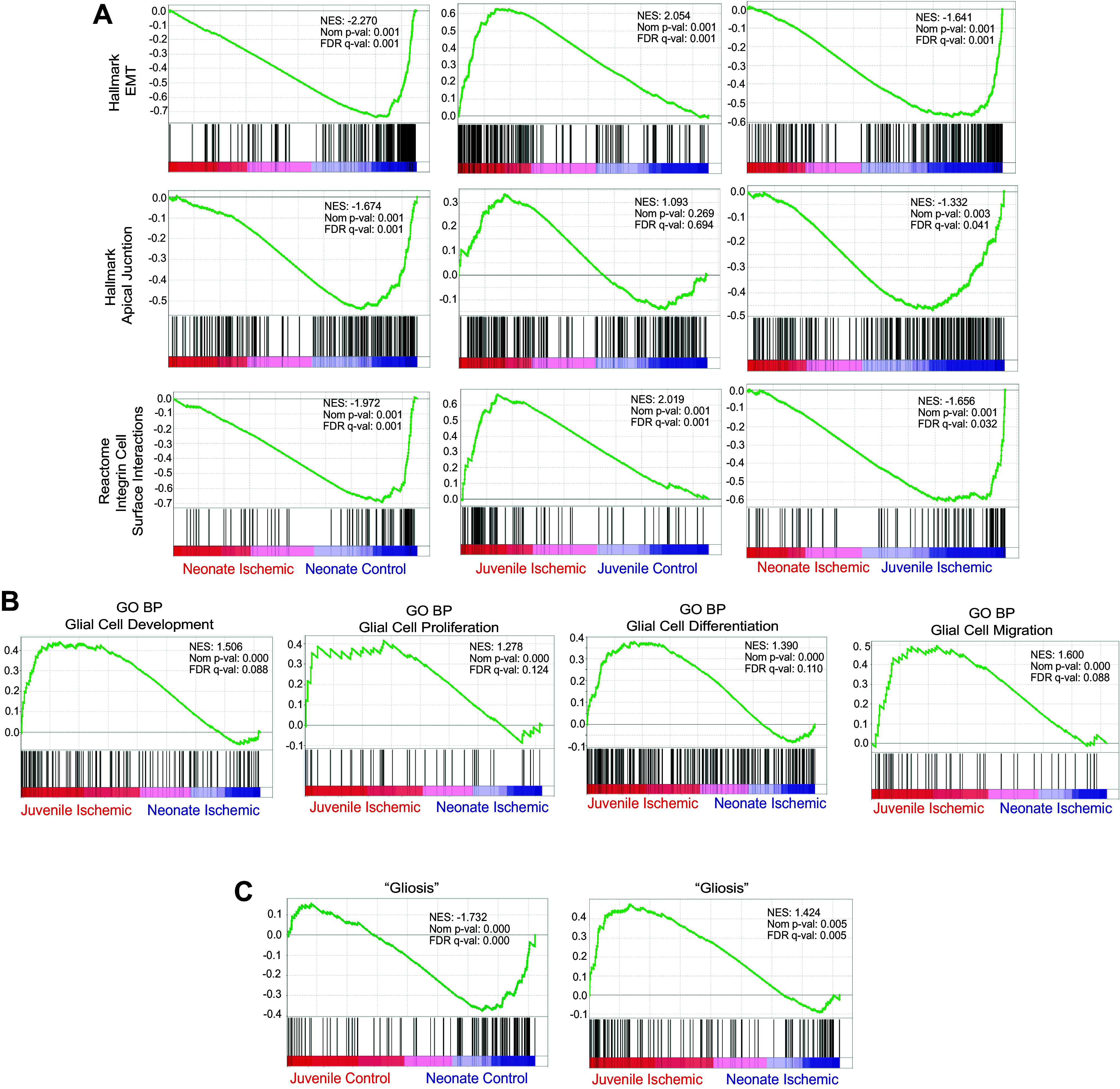
Transcriptomic differential expression analyses identify enrichment for select pathways implicated in epithelial adhesion and migration, and enteric glial cell reactivity termed “gliosis” in juvenile pigs. Gene Set Enrichment Analyses (GSEA) of RNA-sequencing data from control and ischemia-injured neonate and juvenile mucosa. The green line shows transcripts from the specified pathway with increased (positive values) or decreased (negative values) expression by rank. Black bar coding represents the location of individual genes on the green curves. Normalized enrichment score (NES), nominal *P* values, and false discovery rates (FDR) *Q* values are shown on each graph. FDR *Q* value ≥ 0.05 was considered significant. *A*: comparisons of the effect of injury on transcripts within age groups (*left* and *middle*) and the effect of age on injury transcripts (*right*) are shown. Activation of gene sets associated with epithelial migration (Hallmark “Epithelial to Mesenchymal Transition” [EMT], Hallmark “Apical Junction,” and Reactome “Integrin Cell Surface Interactions” are markedly impaired in neonates (*left* and *right* columns). Within juveniles, Hallmark “Epithelial to Mesenchymal Transition” [EMT] and Reactome “Integrin Cell Surface Interactions” are enriched in ischemic mucosa (*middle* column). *B*: within ischemic mucosal samples, Gene Ontology Biological Processes (GO BP) “Glial Cell Development,” “Glial Cell Proliferation,” “Glial Cell Differentiation,” and “Glial Cell Migration” trend toward enrichment in juveniles as compared with neonates. *C*: “Gliosis” as defined by Schneider et al. (32) was enriched in control or ischemic juveniles as compared with control or ischemic neonates, respectively.

Finally, pairwise comparisons allowed the use of Gene Set Enrichment Analysis (GSEA) to evaluate whether postnatal age imparts a significant positive or negative relative enrichment for several Molecular Signatures Database “hallmark” gene sets associated with epithelial cell functions that are relevant to epithelial wound healing in tissues recovering from ischemic injury. Within neonatal mucosa, the hallmark gene sets “epithelial to mesenchymal transition,” “apical junction,” and “integrin-cell surface interactions” were negatively correlated with the ischemic condition (Fig. 3*A*, *left*). Within juvenile mucosa, the opposite correlation was found with two of these gene sets, in that “epithelial to mesenchymal transition” and “integrin-cell surface interactions” were positively correlated with the ischemic condition (Fig. 3*A*, *middle*). When comparing the ischemic condition across age groups, “epithelial to mesenchymal transition,” “apical junction,” and “integrin-cell surface interactions” were negatively correlated with the neonates, enriched in juveniles, consistent with the defective epithelial barrier and defective epithelial restitution observed in this age group (Fig. 3*A*, *right*) (19). These results agree with the cellular phenotypes observed in neonates and juveniles, further demonstrating the lack of a coordinated transcriptional response to epithelial barrier injury in neonates as compared with juveniles.

### Gene Signatures That Drive Development of the Enteric Glial Cell Network and “Gliosis” Response to Injury Are Enriched in Ischemia-Injured Juvenile Mucosa

Because of the predominant role of EGC in promoting epithelial restitution in adults and in light of evidence that mucosal EGC network establishes postnatally in rodents, we used GSEA to compare relative enrichment for gene datasets related to glial cell biology in transcriptomic profiles of ischemic mucosa from juvenile versus neonates (20, 22, 23, 28). Using GSEA, we found that Gene Ontology Network Biological Processes gene sets for “glial cell development,” “glial cell proliferation,” “glial cell differentiation,” and “glial cell migration” demonstrated a strong trend toward enrichment in the juvenile mucosa as compared with the neonatal mucosa (Fig. 3*B*). In many disease states, including ischemia, an EGC may transition to an activated immune phenotype termed “gliosis.” This is characterized by an increased expression of a set of genes that include a subset of inflammatory response genes, and this gene set was defined in a new GO term published by Schneider et al. (32) in 2021. We interrogated this gene set in neonatal and juvenile mucosal tissue from both uninjured and ischemia-injured jejunum using GSEA and, importantly, we found that “gliosis” is enriched in juvenile tissues both at baseline and when undergoing ischemic injury as compared with neonates (Fig. 3*C*). Altogether, these transcriptomic analyses indicate that gene sets that drive development of the enteric glial cell network and “gliosis” response to injury are enriched in ischemia-injured juvenile mucosa, and the relative lack of these gene signatures in neonatal mucosa suggests that reduced EGC network development and activity might play an important role in age-dependent defects in epithelial restitution.

### The Three-Dimensional Density of EGC within the Subepithelial Space Shifts Discernably during Early Postnatal Development

**Figure 4.**
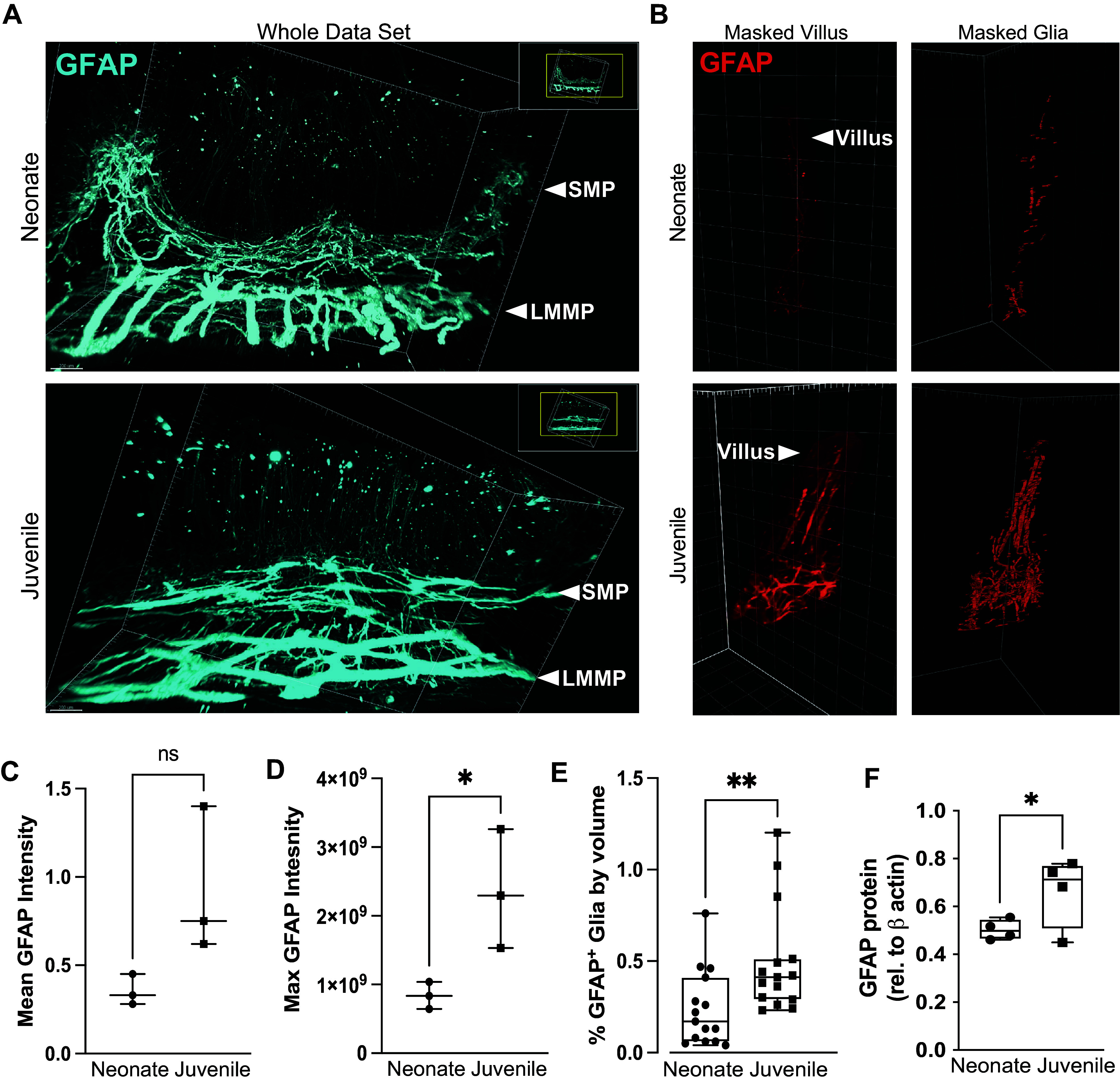
Glial fibrillary acidic protein (GFAP)^+^ enteric glial cell (EGC) density is increased in the subepithelial space of juvenile mucosa as compared with neonates. *A* and *B*: light sheet imaging of GFAP^+^ EGC networks in immunolabeled three-dimensional imaging of solvent cleared organs (iDISCO)-prepared whole jejunum revealed lower glial density in the lamina propria of neonatal jejunum as compared with juveniles. Two-dimensional (2-D) representations of three-dimensional (3-D) EGC networks labeled against glial fibrillary acidic protein (GFAP) in neonatal (*top*) and juvenile (*bottom*) jejunum. Panels in blue represent the entire data set while panels in red represent a single villus masked (*middle*) for EGC volume quantification by custom-designed algorithm using Imaris software (*right*). *C* and *D*: trending increased mean and increased maximum GFAP signal intensity was measured within the lamina propria of juveniles as compared with neonates. *E*: percentage of villus volume occupied by GFAP^+^ glia is higher in juvenile vs. neonates. (Biological *n* = 3 or 4. *P ≤ 0.05 by one-tailed Mann–Whitney *t* test). *F*: increased GFAP protein expression was quantified in the microdissected subepithelial mucosa of juveniles as compared with neonates (*n* = 4. *P ≤ 0.05, **P ≤ 0.01 unpaired Student’s *t* test).

In light of our transcriptomic results implicating the EGC network, we next sought to define age-dependent differences in the EGC network density in the porcine jejunal mucosa. To completely visualize and quantify changes in the three-dimensional network of EGC in the postnatal pig jejunum, we utilized a whole tissue imaging approach called immunolabeled three-dimensional imaging of solvent-cleared organs (iDISCO) in which optically cleared full-thickness sections of intestine are stained with fluorescent antibodies and imaged in three dimensions with a light sheet microscope (36). Full-thickness jejunum samples stained against glial fibrillary acidic protein (GFAP), a component of the cytoskeleton in certain EGC subtypes known to be most important for directly impacting epithelial barrier functions by paracrine signaling in the immediate epithelial space (23, 26, 37–43), demonstrated a dense and complex network of GFAP^+^ EGC throughout the submucosal plexus (SMP, Fig. 4*A*) and longitudinal muscle myenteric plexus (LMMP, Fig. 4*A*) in both neonates and juveniles. In the lamina propria, however, there was a greater density of GFAP^+^ EGC in the immediate subepithelial space of jejunal villi in juveniles as compared with neonates (Fig. 4*B*, *left*). GFAP^+^ EGC were identified by a customized surfaces algorithm in Imaris software (Fig. 4*B*, *right*) and quantification demonstrated that maximum GFAP signal intensity and GFAP^+^ EGC density, measured as the percent volume of the villi occupied by GFAP^+^ EGC, were increased in juvenile lamina propria compared with neonates (Fig. 4, *D* and *E*).

To further quantify changes in GFAP^+^ EGC density within the discrete layers of the jejunum during the postnatal period, we evaluated the levels of expression of GFAP protein present in jejunal mucosa isolated from neonatal and juvenile pigs by microdissection. There was increased GFAP in juveniles as compared with neonates (Fig. 4*F*), in agreement with transcriptomic trends (Fig. 3*B*) and 3-D immunofluorescence quantification (Fig. 4, *D* and *E*). Altogether, these results suggest that the three-dimensional distribution of GFAP^+^ EGC in the immediate subepithelial space, the EGC subtype most implicated in direct paracrine regulation of epithelial barrier function and repair, is enhanced in juvenile-aged jejunal mucosa as compared with neonates.

### Fluoroacetate Inhibits Barrier Restitution after Ischemic Injury in Juveniles

**Figure 5.**
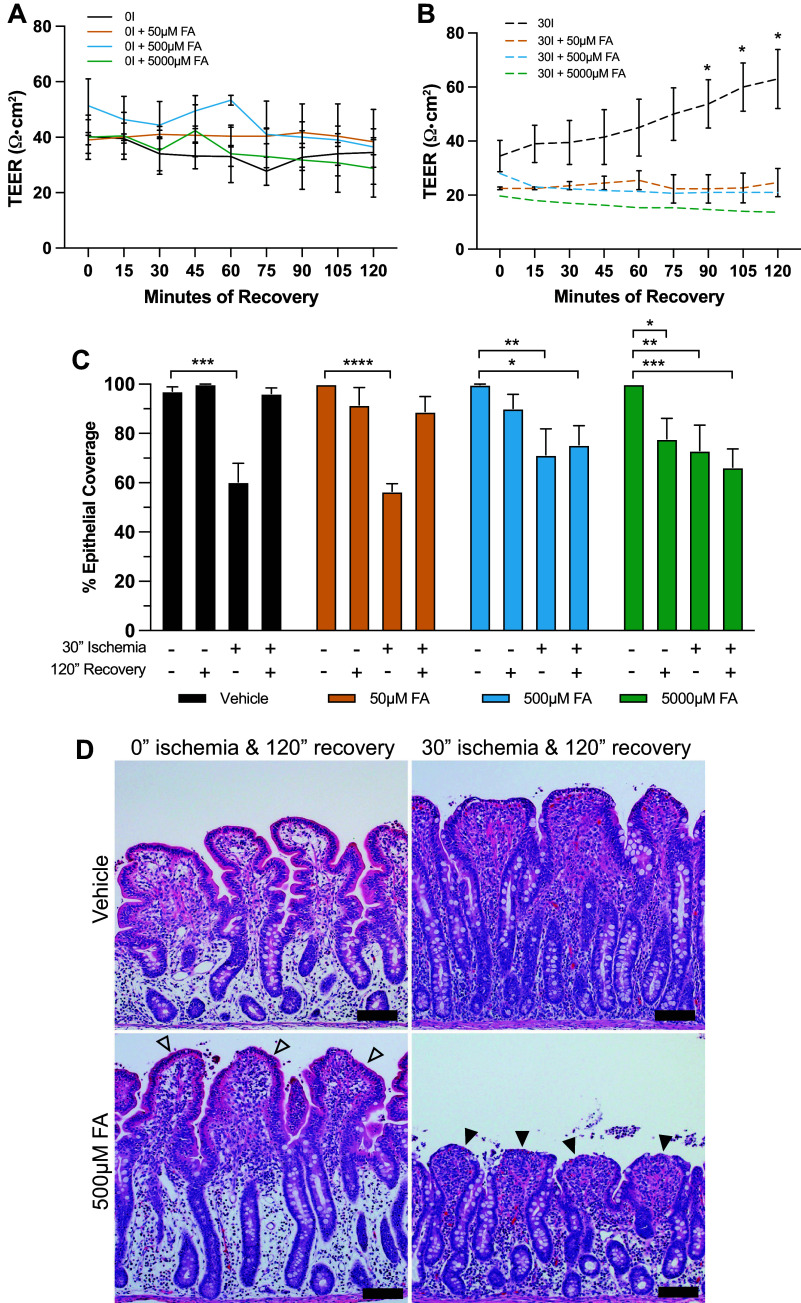
Treatment with 500 µM enteric glial cell (EGC) inhibitor fluoroacetate (FA) inhibits restitution in ischemia-injured juvenile jejunum. *A*: incubation with FA at any dosage did not affect mucosal transepithelial electrical resistance (TEER) in the absence of intestinal ischemic injury (*n* = 3 or 4. *P* = 0.150 for overall effect of treatment on TEER over time by two-way ANOVA). *B*: no recovery of TEER was seen in the presence of FA, regardless of concentration. (*n* = 3 or 4. *P* < 0.0001 for overall effect of treatment on TEER over time by two-way ANOVA. *P < 0.05, **P < 0.01, ***P < 0.001, ****P < 0.0001 by Dunnett’s multiple comparisons test.) *C*: 50 µM FA did not significantly alter epithelialization patterns vs. tissues incubated in standard Ringer solution. FA (5 mM) inflicted significant epithelial loss in the absence of ischemic injury. Without causing significant epithelial loss in the absence of ischemic injury, 500 µM FA inhibited epithelial restitution following ischemia. [*n* = 3 or 4. *P* < 0.0001 for overall effect of ischemia condition on TEER over time and a significant interaction between ischemia condition and treatment (*P* = 0.0223) by two-way ANOVA. *P < 0.05, **P < 0.01, ***P < 0.001, ****P < 0.0001 by Dunnett’s multiple comparisons test. Scale bar = 50 µm.] *D*: representative micrographs showing effect of 500 µM fluoroacetate on epithelial restitution. Note no significant epithelial injury in nonischemic mucosa (open arrowhead) but inhibited restitution in ischemic mucosa (solid arrowhead).

The gliotoxin fluoroacetate (FA) has been used experimentally to inhibit glial cell metabolism and activity, therefore, we sought to utilize FA to inhibit glial cell activity to directly implicate the EGC network on barrier restitution (44–46). To examine whether FA-mediated inhibition of EGC would induce a restitution defect in the repair-competent juvenile pig model, barrier function was examined by both electrophysiology and histomorphometry during ex vivo recovery from 30 min of intestinal ischemia with and without the application of increasing doses of FA. Treatment with any dose of FA during ex vivo incubation did not affect the TEER in uninjured mucosa (Fig. 5*A*) but did inhibit recovery of TEER in juvenile jejunal mucosa injured by 30 min of surgical ischemia (Fig. 5*B*). Histomorphometry revealed significant loss of epithelium at a high dilution of 5,000 µM FA even in the absence of ischemic injury indicating that FA becomes toxic to the epithelium at this dose (Fig. 5*C*, green bars); however, the TEER remains unchanged in this set of tissues (Fig. 5*A*), which suggests that the remaining epithelium is able to compensate with enhanced tight junction activity in the absence of ischemic injury. At a low dilution of 50 µM FA, epithelial coverage was similar to control tissues across all combinations of ischemia and recovery (Fig. 5*C*, yellow and black bars, respectively). The persistent reduction in TEER despite the evidence of effective epithelial restitution at the end of the recovery period in the 50 µM FA group suggests that FA inhibition at this lower dose does not impact restitution but does reduce tight junction closure efficiency when combined with ischemic injury. At a midrange dilution of 500 µM FA, there was no significant loss of epithelium in the absence of ischemic injury, which together with its lack of impact on TEER in uninjured mucosa suggests that this dose is not toxic to the epithelium. This was further validated by testing the effect of 500 µM FA on epithelial restitution in vitro by scratch wound assays in IPEC-J2 cells (Supplemental Fig. S2). However, a key finding was that restitution of epithelial coverage during ex vivo recovery after ischemia injury was inhibited by 500 µM FA, effectively recreating the restitution defect seen in the neonatal model (Fig. 5*C*, blue bars). The histological appearance of nonischemic mucosal epithelium appeared very similar after 120 min ex vivo incubation in Ussing chambers with and without treatment with 500 µM FA. Complete epithelialization was observed at the mucosal surface of villi treated with 500 µM FA (Fig. 5*D*, open arrowheads) providing further evidence of a lack of a nonspecific toxic effect of FA at this dosage on epithelial morphology in uninjured jejunum. After 30 min of intestinal ischemic injury, tissues incubated ex vivo in control conditions demonstrated effective restitution characterized by the flattened morphology of the newly restituted epithelial cells at the villus tips as previously reported, contrasting with the persistent epithelial defects observed in the tissues incubated ex vivo in 500 µM FA, which demonstrated a persistent epithelial defect at the tip of the villi, very similar to previously reported restitution defect in neonates (Fig. 5*D*, closed arrowheads) (19).

Along with other phenotypical parameters, alterations in GFAP and IL-6 in EGC are indicative of a reactive state broadly referred to as “gliosis” in some disease states, including ischemia (32, 47–50). To validate that 500 µM FA treatment alters EGC reactivity in response to ischemic injury in our model, we measured FA-mediated derangement of ischemia-induced changes in GFAP and IL-6 signal intensity in the submucosal EGC. Ganglia of the submucosal plexi were used as a surrogate population for these measurements as these are the GFAP^+^ groups of cells most distinctly visible by immunofluorescent histology and easily outlined for quantification in these experimental tissues. Longitudinal and circular muscle layers and thus the muscular enteric plexi therein were bluntly dissected away before ex vivo incubation for experimentation and so those ganglia were therefore unavailable for imaging. Interestingly, ischemia-induced increases in GFAP signal intensity were blocked by FA (Supplemental Fig. S1, *A* and *B*), whereas FA-induced increased IL-6 intensity in these ganglia (Supplemental Fig. S1, *C* and *D*). Altogether, these results suggest that unaltered and carefully controlled EGC activity is instrumental in orchestrating mucosal restitution in ischemia-injured jejunum of juvenile pigs.

### Coculture with EGC from Muscular and Submucosal Plexuses of Neonatal Jejunum Enhance In Vitro Restitution in Intestinal Epithelial Cells

**Figure 6.**
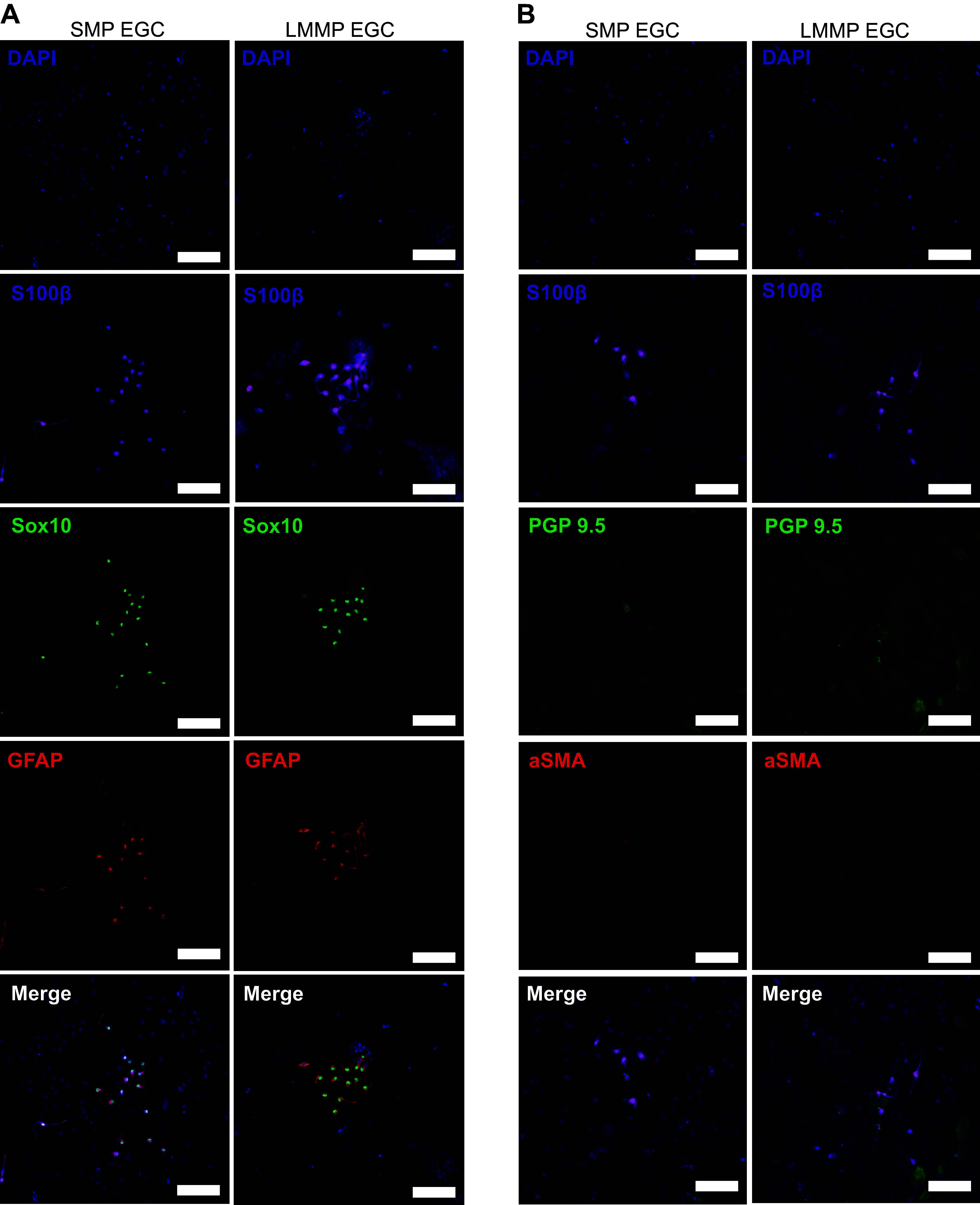
A heterogeneous population of pig enteric glial cell (EGC) can be isolated and maintained in vitro. *A*: primary EGC from pig jejunal submucosal plexus (SMP) and longitudinal muscle myenteric plexus (LMMP) in culture immunolabeled for EGC markers S100β (magenta) Sox10 (green), and glial fibrillary acidic protein (GFAP) (red). Note the small number of negative cells and the fibrillary staining pattern of GFAP and Sox10^+^ nuclei in small subsets of EGC vs. the more diffuse cytoplasmic and nuclear pattern of S100β most of the EGC in culture. *B*: primary EGC from pig jejunal SMP and LMMP in culture immunolabeled for EGC markers S100β (magenta), PGP 9.5 (green), and αSMA (red). Note the very low numbers of PGP 9.5 or αSMA-positive cells. (DAPI nuclear counterstain (blue), Scale bars = 100 µm.)

**Figure 7.**
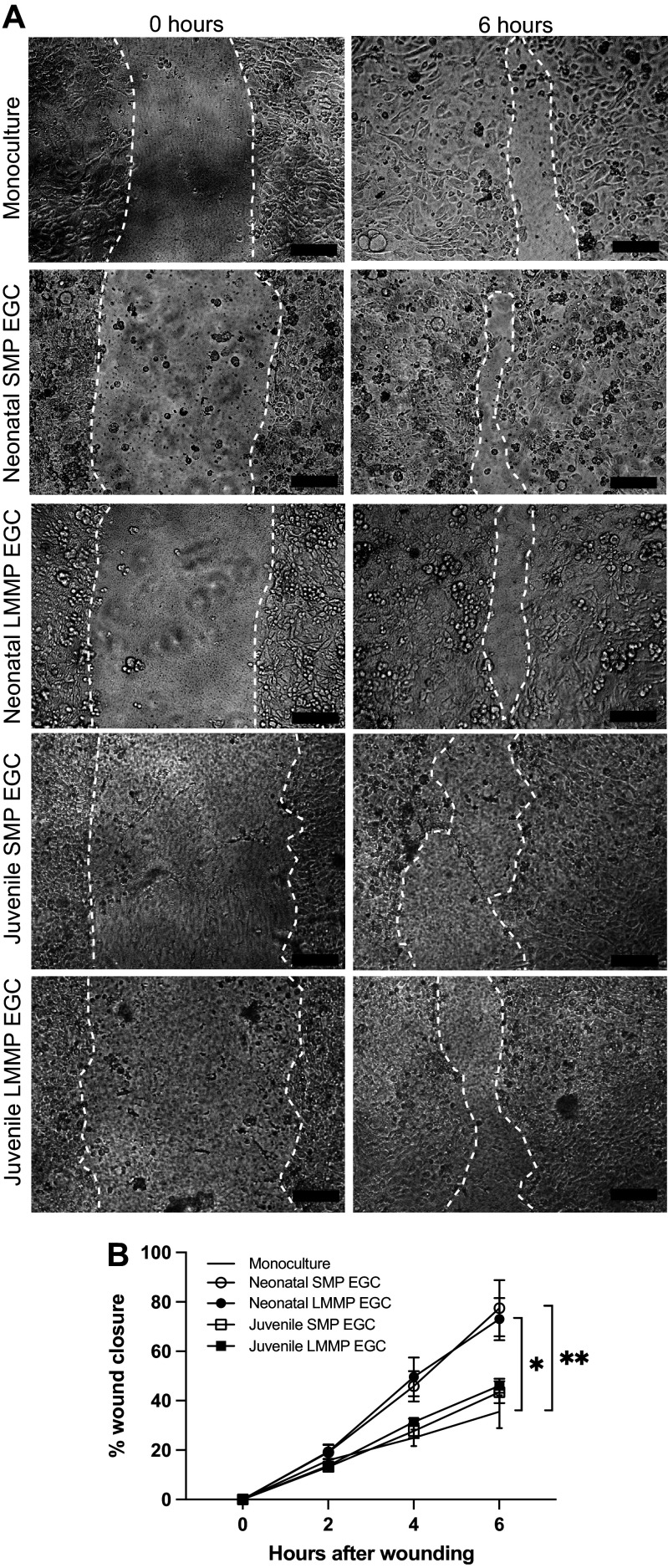
IPEC-J2 monolayers restitute scratch wounds more efficiently when in coculture with neonatal pig enteric glial cell (EGC). *A*: representative photomicrographs of IPEC-J2 monolayers at 0 and 6 h after scratch wounding when in monoculture (left) or in coculture with neonatal or juvenile submucosal plexus (SMP) EGC (middle) or longitudinal muscle myenteric plexus (LMMP) EGC (right). Wound margins are outlined with a broken white line. Scale bars are 100 µm. *B*: quantification of wound closure rates based on wound surface areas at 0-, 2-, 4-, and 6 h after wounding revealed a significant effect of time (*P* < 0.0001) and coculture (*P* = 0.013) and an interaction between time and coculture (*P* < 0.0001) on wound healing was observed by two-way repeated-measures ANOVA (*n* = 5–7). Post hoc testing revealed a significant difference between neonatal SMP coculture vs. monoculture (*P = 0.0105) and between neonatal LMMP coculture and monoculture (**P = 0.0088) at 6-h postwounding (*n* = 5–7).

To examine glial-epithelial interactions more precisely during barrier repair, a coculture model with primary pig EGC was implemented. Primary pig EGC was isolated from the submucosal (SMP) and longitudinal muscle myenteric plexuses (LMMP) of uninjured pig jejunum. Purity of a heterogeneous population EGC culture was confirmed by the expression of the EGC markers GFAP, Sox10, and S100β with very few negative cells present (Fig. 6*A*). Representative images of primary EGC culture showed subpopulations of EGC with Sox10+ nuclei in close association with GFAP^+^ EGC showing a fibrillar arrangement of GFAP-containing cytoskeletal elements, and a more diffuse cytoplasmic and nuclear pattern for S100β signal in most cells observed in culture (Fig. 6*A*). Additional cultures stained against S100β were costained against PGP 9.5 and αSMA as markers of contaminating cells of neuronal and fibroblast origins, respectively, and very few contaminating cells were observed in the heterogeneous EGC cultures (Fig. 6*B*). To examine epithelial restitution in a coculture transwell system, neonatal and juvenile pig jejunal EGC from either the SMP of the LMMP were maintained on the bottom of a well and placed in the presence of IPEC-J2 confluent monolayers seeded on permeable membranes in the apical chamber suspended above the EGC for 24 h to allow glial-epithelial paracrine bidirectional exchanges of soluble factors before manually inflicting a scratch wound (Fig. 7*A*). Six hours after wounding, there was a significant increase in restitution rates in IPEC-J2 monolayers in coculture with pig EGC from either the SMP or LMMP of neonates as compared with IPEC-J2 restitution in coculture with juvenile SMP or LMMP EGC or IPEC-J2 restitution in monoculture. Measured wound closure at 6 h after wounding was 77.4% and 73.0% for neonatal SMP and LMMP, respectively (Fig. 7*B*). These in vitro results provide important proof-of-concept that important prorestitution communications may take place between the epithelium and key subpopulations of EGC when in great enough density and proximity to one another for paracrine signaling interactions to occur and that these EGC subpopulations are highly developmental and dynamic in the early postnatal period.

## DISCUSSION

In combining an in vivo surgical injury model with an ex vivo recovery system, our laboratory has been able to deeply characterize and rescue an age-dependent defect in epithelial restitution in a novel pig model. Ex vivo recovery of injured mucosal tissues in Ussing chambers allows for nearly unlimited technical replicates within one animal, direct electrical and functional measurement of epithelial barrier recovery in real-time and its modulation in response to carefully controlled experimental interventions such as the direct application of homogenized mucosal tissues reported in our 2018 study (19). In the present study, we initially further characterized epithelial restitution after intestinal ischemic injury into later stages of acute mucosal recovery when juvenile tissues have fully recovered barrier function, but neonates have not, to improve our understanding of the epithelial repair defects observed in neonates. This is a strength of the Ussing chamber ex vivo recovery system in that multiple tissue sections may be recovered simultaneously from one animal, which allows for multiple timepoints of recovery to be examined in a paired analysis within one biological replicate. Scanning electron microscopy (EM) imaging confirmed juvenile mucosa exhibits the characteristic epithelial phenotype of restitution, defined by the recruitment membrane from the microvilli into the extension of both lamellipodia and filopodia onto the exposed extracellular matrix of the wound beds. This is highly indicative of successful epithelial migration and wound closure, and indeed, at later stages of acute mucosal recovery, all villi were completely covered with overlapping epithelial cells of this flattened and irregular appearance with some persisting lamellipodial extensions visible. In contrast, neonatal epithelial cells remained round and covered in microvilli throughout the later stages of recovery, without any phenotypic indications of directional migration. This phenotype closely parallels what has been described in other intestinal injury models, such as a punch biopsy murine intestinal wound healing model in which the authors define “wound associated epithelial (WAE) cells” characterized by migratory nature and a shorter height than non-WAE cells, or in vitro scratch-wounding epithelial injury models that describe intestinal epithelial cell spreading and migrating in a similar manner (51–53). These findings substantiate an age-dependent restitution defect defined by the striking contrast in the morphology of newly restituted epithelial cells in juvenile jejunum versus the complete absence of a restitution phenotype observed in wound-adjacent epithelial cells of neonates at later phases of acute barrier repair.

To identify key age-dependent differences in the molecular mechanisms occurring in injured jejunum, we assessed transcriptomic changes occurring in the mucosa of neonates versus juveniles in response to ischemic injury by bulk RNA sequencing. In agreement with the striking observed phenotypic differences, PCA plots of transcriptional expression data from uninjured and injured neonatal and juvenile mucosa suggested a lack of a coordinated transcriptional response in the neonatal mucosa in response to injury whereas juveniles demonstrate distinct transcriptomic profile clustering in response to injury across multiple methods of PCA comparison. These age-dependent differences in coordinated transcriptional responses parallel the age-dependent response in the wound-associated epithelial cells observed by scanning EM and demonstrate a rapid alteration of gene expression in response to injury within a very acute time frame.

To more holistically identify the global signaling mechanisms associated with these transcriptomic changes, we used two complementary analyses, the first one identifying pathways and mechanisms representative in the significantly DE genes (Ingenuity Pathways Analysis, IPA) and the second one testing for the relative enrichment of predefined gene sets in global transcriptomic profiles (Gene Set Enrichment Analysis, GSEA). When comparing injured neonatal mucosa with injured juvenile mucosa to interrogate solely age-dependent differences in transcriptional response to injury, several interesting pathways emerged. Two gene sets important in epithelial adhesion, Hallmark “Apical Junction” and Reactome “Integrin Cell Surface Interactions,” were found to be enriched in ischemia-injured juveniles versus ischemia-injured neonates. Apical junctional complexes contain the zonula adherens and the tight junctions, the latter of which are the molecular structures most integral to sealing the interepithelial spaces and forming the intestinal barrier and are instrumental in intestinal barrier repair (54). Integrins are essential to epithelial cell adhesion to the basement membrane, and their precise regulation is critical to cellular migration during coordinated wound healing events in the intestine, as has been shown with integrin β1 in restituting and hypoxic intestinal epithelium (51, 55). Integrins have been shown to also regulate the activation of various intracellular molecular mechanisms controlling cell functions key to mucosal repair, such as the role of α3β1 integrin in inhibiting Smad7, effectively potentiating TGF-β1-mediated keratinocyte migration during cutaneous wound healing (56). Thus, alterations in integrin signaling at the RNA level suggest potentially important implications in epithelial wound healing. Interestingly, the Hallmark Gene Set “Epithelial to Mesenchymal Transition” (EMT) was also found to be enriched in juveniles versus neonates after injury. EMT is a well-described predominantly transcriptionally based transdifferentiation process that consists of coordinated phenotypic changes in epithelial cells leading notably to reduction of cell-cell adhesion, loss of cell polarity, and adoption of a migratory phenotype, just as is observed in intestinal restitution following ischemic injury in juveniles. This transition has been defined in embryogenesis (*type 1 EMT*), inflammation and organ fibrosis (*type 2 EMT*), and epithelial-origin cancers (*type 3 EMT*) (57). An intestinal epithelial cell’s transition to a non-neoplastic, nonfibrotic migratory state during acute barrier repair may involve very similar signaling pathways as EMT and thus identification of gene patterns associated with this phenotype in this model is validating. Furthermore, pathways identified by IPA listed in Table 1 further substantiate reduced cellular migration-associated signaling in the ischemic neonatal mucosa as compared with ischemic juvenile mucosa, including regulation of cytoskeleton, microtubules, filaments, and cellular protrusions, and more broadly “cell movement” and “migration of cells.” Taken together, EM and RNA-seq data strongly support previous findings demonstrating major defects in epithelial restitution following intestinal ischemia in neonatal versus juvenile pigs (19).

As we have previously reported, the neonatal defect in restitution is rescued by direct application of ischemia-injured jejunal homogenate from juvenile-aged pigs, but the component of this homogenate that drives restitution has not yet been identified (19). A list of the top 25 DE genes, as well as a volcano plot comparing the age-dependent response to ischemic injury, demonstrated several genes associated with responses to intestinal injury such as Ubiquitin D (UBD), and IFNγ-mediated intestinal inflammatory responses such as guanylate binding protein 2 (GBP2) and NOD-like receptor 5 (NLRC5) (58–60). Interestingly, IFNγ-dependent gene modules were also induced in EGCs from patients with inflammatory bowel disease and chemokine interferon-γ-inducible protein (CXCL10) has been shown to be an “early immediate response” by EGC in response to intestinal injury-induced interferon λ signaling (61, 62). Given the emerging and growing appreciation for the critical role of EGC in regulating and relaying signaling events in the intestinal mucosa in both homeostatic and pathologic conditions, we have considered their influence in our model (63–65). Of particular relevance to our studies, many have demonstrated direct interactions between EGC and the intestinal epithelium to direct the maintenance and repair of the intestinal barrier (23, 26–28, 38–43, 66). Indeed, functional studies have provided strong evidence using in vitro systems and in vivo rodent models that EGCs promote barrier function and epithelial restitution after injury in adulthood (23, 26). Taken together, we considered whether the molecules present in jejunal homogenate from ischemia-injured juvenile pigs and contributed to the rescue of restitution in neonates originating from EGC. Supporting this hypothesis, prevailing evidence in rodents suggests that the EGC network continues maturation after birth leading us to posit whether these networks may be underdeveloped in the neonatal condition in the pig model (22, 67, 68). From our transcriptomic analysis, GSEA demonstrated a strong trend toward the enrichment of molecular pathways involved in glial cell development and migration in the juvenile versus neonatal ischemic mucosa. This would suggest that pathways coordinating EGC migration and network development into the mucosa are more active in juvenile mucosa as compared with neonates, which is consistent with a putative role played by the EGC network in the age-dependent defects in epithelial restitution observed during the postnatal period. Three-dimensional volume imaging and Western blot analysis quantify a definite increase in GFAP^+^ EGC density within the subepithelial region of juvenile pig jejunum as compared with neonates. Altogether, these findings are indicative of increased GFAP^+^ EGC density in the subepithelial space, which may reflect increased numbers of GFAP^+^ cells, or increased expression of GFAP in individual cells. These GFAP-expressing subpopulations of EGC located just beneath the intestinal epithelium are the population most relevant to paracrine regulation of the epithelial barrier; GFAP^+^ EGC are implicated repeatedly in the literature as most important for mediating epithelial barrier function and repair and the paracrine signaling by which they perform these functions requires an optimal distance of just a few microns between communicating cells (23, 26, 37–43). Of note, EGC of different phenotypes have been shown to differentially express common EGC markers; at least four described subtypes have been proposed without any single definitive pan-EGC marker yet described (69). The striking heterogeneity of EGC as well as the specific locations of these distinct subtypes is increasingly suspected to result in EGC subtype-specific impact on epithelial cell functions (70). Efforts to fully map postnatal changes in EGC network density and cellular composition within the subepithelial space is an important area for ongoing study.

Although strong evidence exists using in vitro coculture systems and in vivo murine models, to the best of our knowledge this study is the first to report that EGC promotes epithelial restitution after ischemia in a large animal model. Indeed, using FA to alter EGC reactivity in juvenile mucosa, we confirmed that this abrogates barrier function recovery and epithelial restitution in juvenile mucosa recovering from 30-min ischemic injury, mirroring the neonatal restitution defect described by our laboratory in 2018 (19). Further substantiating our notion that EGC activity may be responsible for age-dependent failure of restitution, a “gliosis” gene signature as defined by Schneider et al. in 2021 was found to be enriched in juvenile mucosa both under homeostatic conditions as well as during ischemic injury as compared with neonates. In the central nervous system, gliosis is a nonspecific, universal response of glia to injury, and results from many acute conditions including ischemia and stroke (71). In the gut, gliosis involves enhanced inflammatory signaling in EGC, and it has been reported increasingly in the literature in response to many intestinal diseases and injury states like chemotherapy, inflammation, infection, and motility disorders (32, 39, 61, 72–74). This reactive state is associated with the release of many cytokines and factors that would have direct interactions with the mucosal barrier and, thus, could be an important component of mucosal response to acute injury as seen in this pig model of acute intestinal injury. Acute impairment of enteric glial metabolism with FA has been used to alter specific glial functions that are associated with subtle changes to calcium signaling, disruption of connexin-43 hemichannels, and a transition to reactive glia (44). Incubating tissues with 500 µM EGC inhibitor fluoroacetate (FA) reduces ischemia-induced upregulation of GFAP, one described marker of the EGC gliosis phenotype (32, 44). Interestingly, IL-6 signal intensity in GFAP^+^ submucosal ganglia is increased by exposure to 500 µM FA, beyond the small increase that is seen in response to ischemic injury. This may suggest that a different type or degree of reactive gliosis which is characterized by inflammatory cytokines like IL-6 may be induced by FA, and this is sufficient to derange prorepair EGC-epithelial signaling in our model. Given that McClain and Gulbransen et al. (44) found acute effects of FA on intact systems may be due to both an impairment of calcium signaling and a conversion of glia to a reactive state, future studies to more precisely examine the roles of calcium signaling versus a proinflammatory reactive gliosis and cytokine release in driving epithelial barrier repair would be of interest. Taken together with the changes observed in EGC network development in the immediate subepithelial space, these findings demonstrate clear age-dependent differences in the EGC populations of the neonatal and juvenile jejunum that can carry important implications in this model.

In a complementary approach and to demonstrate proof-of-concept more fully, an in vitro scratch wound model of intestinal barrier injury and repair using coculture of pig intestinal epithelial cell monolayer with jejunal EGC from either the SMP or LMMP of neonatal pigs, but not juvenile pigs, demonstrated twofold enhanced restitution efficiency. In our in vitro model, neonatal EGC from the submucosal and deeper regions of the jejunum can enhance epithelial barrier restitution by secreted factors that readily diffuse through cell culture media. Feasibly, this indicates an efficient prorepair paracrine signaling mechanism is present in neonatal EGC that are in the deeper layers of the jejunum, but that these subtypes of cells are too sparse in the immediate subepithelial space to efficiently stimulate epithelial restitution in situ during this early postnatal period. Interestingly, EGC isolated from those same deep tissue layers in juvenile pigs do not seem to impart this in vitro capability which may be due to the already completed migration of this key subpopulation out of these deeper plexuses and into the subepithelial space where we are presently unable to isolate them for in vitro study. Indeed, lineage tracing experiments have elegantly demonstrated that the mucosal EGC network establishes from EGC migrating along a serosa-mucosa axis, i.e., originating from the immediate underneath layer, the submucosal plexus, and this seems to be also true for this pig model (22, 75). Perhaps, this signaling observed in EGC from the deeper layers of the still-developing neonatal jejunum is effective in driving intestinal epithelial repair once they migrate into subepithelium and can approximate the density achieved in this reductive culture system presented here. These data are the first to demonstrate this effect with primary cells from a complex large animal model with high translation potential.

Altogether, our results further refine our model of postnatal age-dependent defects in epithelial repair characterized by a failure in neonates to activate both transcriptional and ultrastructural epithelial restitution phenotypes following ischemia. These data provide key evidence that the presence of a functional population of GFAP^+^ EGC in close proximity to the damaged epithelium plays a critical role in promoting epithelial barrier repair, and thus a suboptimal density of a mature subepithelial EGC network in neonates may be contributing to the observed age-dependent defect in epithelial barrier restitution. Limitations of these studies include the inability to account for certain host inputs in these reductionist ex vivo and in vitro models, which should be addressed in future studies to test these findings in whole animal in vivo systems. Ongoing work to fully characterize the postnatal development, distribution, and function of key EGC subtypes and to understand age-dependent EGC-mediated paracrine signaling in this pig model, and validating key findings in vivo, has the potential to drive translational efforts to develop novel therapeutic strategies to improve outcomes in neonates suffering from intestinal failure associated with epithelial barrier disruption.

## DATA AVAILABILITY

Data will be made available upon reasonable request.

## SUPPLEMENTAL MATERIAL

10.6084/m9.figshare.24018543.v1 Supplemental Figs. S1 and S2: https://doi.org/10.6084/m9.figshare.24018543.v1.

## GRANTS

This study was supported by National Institutes of Health (NIH) Grants K01 OD 028207 (to A.L.Z.), NIH-NICHD R01 HD095876 (to A.T.B., J.O., and L.V.L.), and P30 DK034987 (to A.T.B.) and US Department of Agriculture-National Institute of Food and Agriculture Grants 2019-67017-29372 (to A.T.B., J.O., and L.V.L.).

## DISCLOSURES

No conflicts of interest, financial or otherwise, are declared by the authors.

## AUTHOR CONTRIBUTIONS

A.L.Z., E.A.H., A.E.S., M.S.T., S.T.M., J.O., L.V.L., and A.T.B., conceived and designed research; A.L.Z., M.L.C., E.A.H., A.E.S., M.S.T., and T.A.P. performed experiments; A.L.Z., M.L.C., S.E.C., E.A.H., A.E.S., T.A.P., L.V.L., and A.T.B. analyzed data; A.L.Z., M.L.C., S.E.C., E.A.H., A.E.S., M.S.T., S.T.M., J.O., L.V.L., and A.T.B. interpreted results of experiments; A.L.Z., M.L.C., S.E.C., and E.A.H. prepared figures; A.L.Z. and E.A.H. drafted manuscript; A.L.Z., M.L.C., S.E.C., E.A.H., T.A.P., S.T.M., J.O., L.V.L., and A.T.B. edited and revised manuscript; A.L.Z., M.L.C., S.E.C., E.A.H., A.E.S., T.A.P., S.T.M., J.O., L.V.L., and A.T.B. approved final version of manuscript.
